# Editorial: Positive Educational Approaches to Teaching Effectiveness and Student Well-being: Contemporary Approaches and Guidelines

**DOI:** 10.3389/fpsyg.2022.1015064

**Published:** 2022-09-26

**Authors:** Matthew Cole, Rebecca Shankland, Mirna Nel, Hans Henrik Knoop, Sufen Chen, Llewellyn Ellardus van Zyl

**Affiliations:** ^1^College of Business and Information Technology, Lawrence Technological University, Southfield, MI, United States; ^2^Laboratoire DIPHE (Développement, Individu, Personnalité, Handicap, Education), Université Lumière Lyon 2, Lyon, France; ^3^Institut Universitaire de France, Paris, France; ^4^Optentia Research Unit, North-West University, Vanderbijlpark, South Africa; ^5^Department of Education, Aarhus University, Aarhus, Denmark; ^6^Graduate Institute of Digital Learning and Education, National Taiwan University of Science and Technology, Taipei, Taiwan; ^7^Department of Industrial Engineering and Innovation Sciences, University of Eindhoven, Eindhoven, Netherlands; ^8^Department of Human Resource Management, University of Twente, Enschede, Netherlands; ^9^Department of Social Psychology, Institut für Psychologie, Goethe University, Frankfurt am Main, Germany

**Keywords:** positive education, positive psychology, wellbeing, academic performance, teaching effectiveness

## Introduction

“In the thrall to content and qualifications, we have forgotten the deeper purpose of education. In the rush to make young people into successful exam passers, we have overlooked their deeper need to become successful people” (Claxton, 2008, p. ix). Positive education is a growing area of educational research that integrates elements of positive psychology with educational practices to promote the mental health and wellbeing of students as fundamental to student success (Seligman et al., 2009; Van Zyl et al., 2022). Positive education programs in schools encompass empirically validated and scientifically informed interventions, proactive strategies and systems thinking to enable a shared purpose toward student wellbeing (Kern et al., 2020; Van Zyl, 2021). Interest in positive education has increased over the past decade as the focus of academic success is no longer only on academic performance (Krifa et al., 2022). According to key international associations, education policy makers, teachers and parents should prioritize student mental health and help students maximize wellbeing (OECD, 2017; WHO and UNESCO, 2021). Positive education includes interventions from positive psychology that target student mental health and emphasize student wellbeing as an essential educational outcome by promoting resilience, self-efficacy, strengths, capabilities, and other non-cognitive skills (International Positive Education Network, 2017; Halliday et al., 2020; Van Zyl et al., 2021; Van Zyl and Rothmann, 2022). Furthermore, the academic, financial, and health stressors arising from the COVID-19 pandemic (Krifa et al., 2021; Frenzel et al., 2022), and a growing social focus on diversity, equity, and inclusion (Govorova et al., 2020; Van Zyl and Salanova, 2022) highlight the need for ongoing positive education research on student wellbeing. This special issue/Research Topic identified current innovative approaches, tools, interventions, methodologies, models and guidelines in positive education.

## Structure and contribution of the Research Topic

The manuscripts in this Research Topic, summarized in Table 1, are classified into three sections: review papers, survey design papers, and intervention papers.

**Table 1:** Summary of contributions to the Research Topic.

**No**	**Author**	**Title**	**Purpose**	**Views**	**Citations**
**Review papers**					
1	Chafouleas and Iovino	Engaging a Whole Child, School, and Community Lens in Positive Education to Advance Equity in Schools	The aim of this review paper was to frame a positive education approach as necessary to advance equitable opportunities in education due to its integration of (a) developmental systems theory, (b) prevention science, (c) ecological systems theory, and (d) implementation science.	5,440	3
2	Iovino et al.	Teaching Simple Strategies to Foster Emotional Well-Being	The aim of this paper was to identify strategies used in cognitive behavioral therapy (CBT) interventions that can be proactively and easily used in schools to promote emotional wellbeing (EWB) in youth who may be experiencing anxiety and depression. The identified strategies were self-awareness, self-soothing, and social relationships.	3,920	0
3	Waters and Loton	Tracing the Growth, Gaps, and Characteristics in Positive Education Science: a Long-Term, Large-Scale Review of the Field	This large-scale quantitative review used publication data to track the presence of positive education terms over a 100+ year period across 35 psychology journals and education journals utilizing two analytical methods.	3,239	3
**Survey design papers**					
4	Baatouche et al.	Meaning of Education and Wellbeing: understanding and Preventing the Risk of Loss of Meaning in Students	The aim of this paper was to analyze and understand, from an existential perspective, the psychological resources that students can activate in order to attribute meaning to their training.	978	0
5	Chhajer and Chaudhry	What Makes Indian Management Students Thrive? Role of Decision-Making Discretion, Broad Information Sharing, and Climate of Trust	This paper examines (a) the relationship of decision-making discretion, broad information sharing and climate of trust with thriving, and (b) the role of self-determination theory in determining this relationship.	808	0
6	Donohue and Bornman	Academic Well-Being in Higher Education: a Cross-Country Analysis of the Relationship Between Perceptions of Instruction and Academic Well-Being	The purpose of this research was to explore the relationship between U.S. and South African university students' perceptions of the overall quality of instruction (PQI) they experienced since COVID-19 and their academic wellbeing.	844	0
7	Li et al.	The Association Among Achievement Goal Orientations, Academic Performance, and Academic Wellbeing Among Chinese Medical Students: A Cross-Sectional Study	This study aimed to explore the status of achievement goal orientations among medical students in China and to further identify the association among academic performance, academic wellbeing, and achievement goal orientations.	2,125	2
8	Lillard et al.	An Association Between Montessori Education in Childhood and Adult Wellbeing	The study aimed to evaluate the Montessori pedagogy as a means to enhance wellbeing contemporaneously and predictively, including self-determination, meaningful activities, and social stability.	11,925	4
9	Moosa and Bekker	Working Online During COVID-19: Accounts of First Year Students Experiences and Well-Being	The aim of this paper is to explore how inclusive policy, practice and culture at one public South African university supported students' wellness while learning online during emergency remote learning.	1,181	0
**Review papers**					
10	Utvær et al.	Nursing Students' Emotional State and Perceived Competence During the COVID-19 Pandemic: the Vital Role of Teacher and Peer Support	The aim of this study was to investigate the associations between peer support, teacher support, emotional state, and perceived competence in nursing students during the pandemic.	2,480	0
11	van Dijk et al.	Experiences of Clinical Clerkship Students With Mindfulness-Based Stress Reduction: a Qualitative Study on Long-Term Effects	The purpose of this study was to explore the mindfulness practice, its long-term effects, facilitators and barriers, in clinical clerkship students 2 years after participation in an 8-week mindfulness-based stress reduction (MBSR) training.	908	1
12	Van Tonder et al.	Enabling Self-Directed Academic and Personal Wellbeing Through Cognitive Education	The aim of this study was to explore the effectiveness of an intervention initiative that exposes teachers to foregrounding Cognitive Education to establish the latent potential of the intervention for assisting learners to develop self-regulating abilities that progressively inspires increased self-directed action.	1,449	0
13	White et al.	Unintended Positive Consequences of Development Centers in University Graduates	The aim of this study was to identify whether the generalized self-efficacy of graduates can be positively affected by a development center approach in the short-term and long-term.	841	0
14	Zalazar-Jaime et al.	Contribution of Academic Satisfaction Judgments to Subjective Well-Being	This paper examined the contribution of academic satisfaction (AS) judgments on subjective wellbeing (SWB) in university students.	430	0
**Intervention papers**					
15	Berg et al.	The Development of Teachers' and Their Students' Social and Emotional Learning During the “Learning to Be Project”-Training Course in Five European Countries	The present study evaluates teachers' and their students' readiness for social and emotional learning (SEL) during an intervention providing teachers' with skills to teach and assess social and emotional learning in the classroom in five European countries.	3,364	2
16	Zaharia et al.	Proof of Concept: a Brief Psycho-Educational Training Program to Increase the Use of Positive Emotion Regulation Strategies in Individuals With Autism Spectrum Disorder	The present study aimed at testing the practical potential and the preliminary effects of a brief novel psycho-educational training program on positive emotion regulation for individuals with autism spectrum disorders.	1,758	0

### Review papers

Chafouleas and Iovino present a conceptual review that a positive education approach must be embedded within a whole child, school, and community lens to advance equity in schools. Such an approach is theory-driven with explicit integration across bodies of science. The authors first provide a brief overview of schools as a context to serve as assets or risks to equity, followed by a discussion of theory and science using a whole child, whole school, and whole community lens. They contend positive education will advance equity when grounded in integrated theory and science across developmental systems theory, prevention science, ecological systems theory, and implementation science.

The review paper by Iovino et al. presents results from among 35 papers on evidence-informed strategies that can be used in schools to promote positive feelings in the moment and build coping behaviors that facilitate tolerance of uncertainty. They focus on those strategies that educators can easily and routinely use across ages, stages, and activities. Selected strategies are primarily tied to cognitive behavioral theory, broadly organized across categories of self-awareness, self-soothing, and social relationships.

The large-scale review by Waters and Loton of 98,571 abstracts from 35 specific journals targeting positive psychology across a 112-year period used computer-aided linguistic analysis and human coding to track the presence of positive education terms. Results of computer-generated linguistic work count analysis identified wellbeing, satisfaction, and the word stems motivate and engage as the most prevalent terms since 2009. In-depth human coding of a subset of positive education abstracts (*n* = 2,805) by a team of five researchers enabled to identify trends pertaining to how positive education research has been conducted in terms of paradigms, designs, methods, tools, samples, and settings from 1950 to 2016. College students and students in secondary school make up the most common samples, with little research in the early childhood years. Quantitative, cross-sectional studies using self-report surveys have been the most common design and method used over the past six decades, suggesting room for growth in qualitative methods and the need for greater longitudinal and intervention designs. The human coding was also used to classify positive education variables into broader categories of research. Nine categories were identified: positive functioning; wellbeing; ill-being; strengths; agency; connection and belonging; identity and personality; school climate and outcomes; and demographics.

### Survey design papers

In exploring the psychological wellbeing of two cohorts of undergraduate and graduate students in France (*N* = 48) using a mixed methods approach, Baatouche et al. combined qualitative interpretative phenomenological analysis (IPA) interview data on lived experiences with quantitative survey data on the meaning of life and the meaning of education. Results suggest seeking the meaning of an education through reflective guidance will increase students' sense of psychological wellbeing by promoting more authentic choices.

Chhajer and Chaudhry focused on thriving, and its critical role in sustaining physical health, positive workplace behavior and wellbeing in 512 Indian management students across five survey design studies. Results found thriving was positively related to decision-making discretion (DMD), broad information sharing (BIS), and climate of trust (COT), with the competence dimension of self-determination theory (SDT) acting as a mediating variable.

Survey data from 889 U.S. and 181 South African undergraduates were analyzed in Donohue and Bornman investigation of the relationship between perceived quality of instruction (PQI) and academic wellbeing as moderated by household income and the cultural value of power distance (PD). Results of regression analysis found PQI is a positive predictor of academic wellbeing for U.S. students regardless of income and PD; students from South Africa were found to have higher wellbeing when they had low PD, regardless of income when PQI was low, but low PD did not associate with academic wellbeing when PQI was high if students were middle- or high-income.

In the study by Li et al., a cross-sectional survey design was conducted in 3,511 medical students from medical colleges in the Harbin, Jiamusi, Mudanjiang, and Qiqihar regions of China. Results of linear regression found medical students' mastery goals were negatively associated with academic stress and positively related to learning adaptability, sleep quality, and subjective wellbeing. A harmonious competitive environment for medical students to improve their academic wellbeing and performance by boosting positive achievement goal orientations is recommended.

Lillard et al. surveyed 834 students who attended at least 2 years of Montessori schools and 1,071 students who attended conventional schools in US and Canada (current mean age of participants was 37 years). Results of structural equation modeling found attending Montessori for at least two childhood years was associated with significantly higher adult wellbeing in terms of general wellbeing, engagement, social trust, and self-confidence.

Moosa and Bekker used a phenomenological qualitative approach to explore the lived experiences of 187 first year South African university students while working online during the COVID-19 pandemic through written responses to four open ended questions. Results highlighted that online learning had a negative effect on student overall spiritual, physical, emotional and social wellness.

The study by Utvær et al. investigated the associations between peer support, teacher support, wellbeing, and perceived competence in 329 nursing students at a large university in Norway during the pandemic. Results of structural equation modeling found that teacher and peer support are significant to nursing students' emotional states and perceived competence.

In the van Dijk et al. study, thematic analysis of interview data from 16 clinical clerkship students from the Radboudumc in Nijmegen, Netherlands 2 years after participation in an 8-week mindfulness-based stress reduction (MBSR) training found interviewees were still engaged in (mostly informal) mindfulness practice contributing to both personal and professional changes. In light of the high clerkship demands, MBSR training could be a valuable addition to medical curricula, supporting medical students in developing necessary competencies to become well-balanced professionals.

Van Tonder et al. explored interview data from 17 pre-school and primary teachers from public and private schools in South Africa who completed an 80-h strengths-based cognitive education intervention. Results found the intervention holds benefits for equipping teachers with teaching strategies to create classroom conditions that nurture the development of thinking skills and dispositions that are important for self-regulating, and ultimately self-directing academic and personal wellbeing.

The study by White et al. combined self-report survey and interview data from 17 industrial psychology graduate students at a select university in the Western Cape, South Africa, to investigate the effect of a development center-based competency assessment intervention on self-efficacy. Results found a positive increase in the general self-efficacy levels of graduates who completed the intervention. This effect was maintained at 3-month follow up testing.

Finally, Zalazar-Jaime et al. analyzed survey data on academic satisfaction (AS), life satisfaction, postive/negative emotions and subjective wellbeing (SWB), from 326 university students (mean age 22 years). Results of structural equation modeling found the positive effects of AS on SWB are partially mediated by life satisfaction judgments and a balance of positive/negative emotions.

### Intervention papers

The study by Berg et al. evaluated the efficacy of an experimental project called “Learning to Be: Development of Practices and Methodologies for Assessing Social, Emotional and Health Skills within Education Systems” which brought together education authorities, teaching practitioners and researchers from seven European countries: Finland, Italy Latvia, Lithuania, Portugal, Slovenia, and Spain. The intervention group consisted of 243 teachers and 2,552 students; the comparison group consisted of 159 teachers and 1,730 students. Results of repeated measures ANOVA found no effect in teachers for the SEL intervention, however, the younger students (8–11 years) were found to have improved self-management skills that were more difficult for the teenagers (12–15 years). This may indicate that positive education interventions should be started before the stormy phase of puberty.

In the study by Zaharia et al., thirty male participants with autism spectrum disorder (aged 10–35 years; training group = 14, waitlist group = 16) underwent a three-session program on the use of adaptive positive emotion regulation (ER) strategies (i.e., attentional deployment, cognitive change, and response modulation). Results of repeated measures MANOVAs and multilevel modeling found the training group showed a significant increase in the self-reported use of the ER strategies compared to the waitlist group. The increase in the use of ER strategies maintained up to 7 weeks in the overall sample. Having reached high satisfaction rates and the intended effects in this proof of concept study, this novel program represents a promising tool to support ER. Future research should next investigate the efficacy of the intervention on day-to-day emotional experience and wellbeing.

## Conclusion

Research supporting students' wellbeing and academic success are closely linked (Dix et al., 2020; Van Zyl et al., 2022), suggesting the importance of fostering wellbeing in the educational context (Ciarrochi et al., 2016; OECD, 2017; Govorova et al., 2020). The manuscripts featured in this Frontiers Research Topic show the many avenues of positive education for the development of positive psychology interventions within educational settings designed to increase student wellbeing and success. Specifically, the papers presented how positive education can enable wellbeing from a sustained whole child, school, community, and equitable lens. As schools transition to a post-COVID world, future research should continue to study these avenues of positive education for building wellbeing in all students.

## Author contributions

MC drafted the first version of the editorial. LZ and RS revised the manuscript. All authors provided input and accepted the final version of the manuscript.

## Conflict of interest

The authors declare that the research was conducted in the absence of any commercial or financial relationships that could be construed as a potential conflict of interest.

## Publisher's note

All claims expressed in this article are solely those of the authors and do not necessarily represent those of their affiliated organizations, or those of the publisher, the editors and the reviewers. Any product that may be evaluated in this article, or claim that may be made by its manufacturer, is not guaranteed or endorsed by the publisher.
